# Selection of euploid blastocysts for cryopreservation with array comparative genomic hybridization (aCGH) results in increased implantation rates in subsequent frozen and thawed embryo transfer cycles

**DOI:** 10.1186/1755-8166-6-32

**Published:** 2013-08-09

**Authors:** Zhihong Yang, Shala A Salem, Xiaohong Liu, Yanping Kuang, Rifaat D Salem, Jiaen Liu

**Affiliations:** 1 ART and PGD Program, Pacific Reproductive Center, Torrance, CA, USA; 2 IVF Division, Beijing Jia En De Yun Hospital, Beijing, People’s Republic of China; 3 Department of Assisted Reproduction, Ninth People’s Hospital, School of Medicine, Shanghai Jiao Tong University, Shanghai, People’s Republic of China

**Keywords:** aCGH, Trophectoderm biopsy, Cryopreservation, Implantation

## Abstract

**Background:**

In assisted reproductive treatments, embryos remaining after fresh embryo transfer are usually selected for cryopreservation based on traditional morphology assessment. Our previous report has demonstrated that array comparative genomic hybridization (aCGH) screening for IVF patients with good prognosis significantly improves clinical and ongoing pregnancy rates in fresh embryo transfer cycles. The current study further investigates the efficiency of applying aCGH in the selection of euploid embryos for cryopreservation as related to pregnancy and implantation outcomes in subsequent frozen embryo transfer (FET) cycles.

**Methods:**

First-time IVF patients with good prognosis undergoing fresh single embryo transfer and having at least one remaining blastocyst for cryopreservation were prospectively randomized into two groups: 1) Group A patients had embryos assessed by morphology first and then by aCGH screening of trophectoderm cells and 2) Group B patients had embryos evaluated by morphology alone. All patients had at least one blastocyst available for cryopreservation after fresh embryo transfer. There were 15 patients in Group A and 23 patients in Group B who failed to conceive after fresh embryo transfer and completed the FET cycles. Blastocyst survival and implantation rates were compared between the two groups.

**Results:**

There were no significant differences in blastocyst survival rates between Group A and Group B (90.9% vs. 91.3%, respectively; *p* >0.05). However, a significantly higher implantation rate was observed in the morphology assessment plus aCGH screening group compared to the morphology assessment alone group (65.0% vs. 33.3%, respectively; *p* = 0.038). There was no miscarriage observed in Group A while a 16.7% miscarriage rate was recorded in Group B (0% vs. 16.7%, respectively; *p* >0.05).

**Conclusions:**

While aCGH screening has been recently applied to select euploid blastocysts for fresh transfer in young, low-risk IVF patients, this is the first prospective study on the impact of aCGH specifically on blastocyst survival and implantation outcomes in the subsequent FET cycles of IVF patients with good prognosis. The present study demonstrates that aCGH screening of blastocysts prior to cryopreservation significantly improves implantation rates and may reduce the risk of miscarriage in subsequent FET cycles. Further randomized clinical studies with a larger sample size are needed to validate these preliminary findings.

## Background

In the procedure of in vitro fertilization, selection of blastocysts for transfer or cryopreservation is routinely conducted on the basis of morphology assessment [1,2]. Since morphological evaluation alone cannot exclude the potential for chromosomal errors in the selected embryos, the transfer or cryopreservation of morphologically “normal-looking” embryos without aneuploidy screening carries unavoidable risk [3-6]. Numerous studies on human oocytes and embryos derived from IVF cycles have shown that aneuploidy is the most common abnormality [7-13], and the high percentage of chromosomal abnormalities contributes substantially to poor reproductive outcomes and miscarriages observed in fertility treatments [3,5,14]. As other investigators have reported [15-19], screening embryos by fluorescence in situ hybridization (FISH) was a preliminary solution to this problem, but the approach was very limited since it could only screen 5 to 12 chromosomes in each embryo. Conventional comparative genomic hybridization (CGH) was then introduced to screen all 23 pairs of human chromosomes with some success [20-23]. More recently, array comparative genomic hybridization (aCGH) [5,6,24-30], single nucleotide polymorphism (SNP) array [31-35] and PCR-based comprehensive chromosomal screening (CCS) [36] have been applied to screen embryos before transfer in order to improve the efficiency of IVF treatments. These techniques enable fresh or frozen embryo transfer so that only euploid embryos are selected for transfer or freezing, thus improving pregnancy and implantation outcomes [5,6,26-28,30,34,36]. However, since experience of using the molecular cytogenetic tests in reproductive medicine is still limited, there is an urgent need to evaluate embryo selection techniques before such technology enters the clinical mainstream [3,6,14,34,35,37,38]. While the optimal genome-wide molecular test for determining the chromosomal status of human embryos remains unsolved [35], there has been very limited information concerning how such technology might affect embryo cryopreservation as related to pregnancy and implantation outcomes in subsequent FET cycles of young, low-risk IVF patients [39]. Therefore, this prospective study is aimed at investigating the impact of aCGH screening on pregnancy and implantation outcomes in subsequent FET cycles of IVF patients with good prognosis.

## Methods

### Patient enrollment and inclusion criteria

First-time IVF patients at our clinics in USA and PR China were recruited in this Institutional Review Board Services (IRB Service) approved prospective study. A written informed consent was obtained from all study participants and all patients received counseling about aCGH screening and cryopreservation. The inclusion criteria include: 1) first time IVF treatment, 2) under 35 year old, 3) no history of miscarriage, 4) presence of both ovaries, 5) normal karyotype, 6) normal endometrial contour, 7) day 2 serum FSH <10 IU/l and estradiol <60 pg/ml. The eligible patients were randomized into two groups: Group A patients (*n* = 55) had their embryos assessed by morphology criteria plus aCGH testing and Group B patients (*n* = 48) had their embryos evaluated by morphology assessment alone. No patients in this study had embryos assigned to both cohorts. All patients had at least one blastocyst available for cryopreservation after fresh embryo transfer. The frozen and thawed embryo transfer was carried out in those patients who failed to conceive after fresh embryo transfer; 15 patients in Group A and 23 patients in Group B completed the FET cycles.

### Ovarian stimulation, oocyte fertilization, embryo culture and evaluation

Prior to ovulation induction, all patients underwent transvaginal ultrasound evaluation with re-measurement of serum FSH, LH and estradiol on day 3 of the index cycle. Pituitary down-regulation was realized with GnRH agonist (GnRH-a, Decapeptyl) on day 21 of the cycle and followed by recombinant FSH (GONAL-f, Merck Sereno; Puregon, Organon) for ovarian stimulation as previously described [6,39,40]. Transvaginal ultrasound and serum estradiol measurements were periodically performed to monitor follicular growth and thickness of endometrial lining. Periovulatory hCG was administered by subcutaneous injection of recombinant hCG (Ovidrel®, Merck Serono, Geneva, Switzerland) when at least 3 follicles reached ≥19 mm in diameter. Oocyte retrieval was conducted by transvaginal ultrasound-guided aspiration 35–36 hours after hCG administration. Oocytes were fertilized with ICSI and fertilization was confirmed with presence of two pronuclei (PN) and two polar bodies (PB) 16 to 18 hours post ICSI. Embryos were cultured to blastocyst stage in G1/G2 sequential media (Vitrolife, Göteborg, Sweden).

In both study groups, blastocysts were evaluated and scored from 1 to 6 on the basis of degree of blastocyst expansion and hatching status as described elsewhere [1,2]. Grade 1 = an early blastocyst with a blastocoele less than half of the embryo volume; Grade 2 = an intermediate blastocyst with blastocoele at least half of the embryo volume; Grade 3 = a full blastocyst with a blastocoele completely filling the embryo; Grade 4 = an expanded blastocyst with a blastocoele larger than the full blastocyst and thinning zona pellucida (ZP); Grade 5 = an hatching blastocyst with herniation of trophectoderm cells from the ZP; and Grade 6 = an hatched blastocyst with a blastocyst completely escaped from the ZP. For blastocysts of grades 3 to 6, the inner cell mass (ICM) was graded as follows: A = many ICM cells packed together tightly; B = several ICM cells grouped loosely and C = very few ICM cells. The trophectoderm (TE) was graded as follows: A = many TE cells forming a cohesive epithelium; B = few TE cells forming a loose epithelium and C = very few large TE cells.

### Blastocyst biopsy and aCGH testing

In the morphology assessment plus aCGH screening (Group A), blastocyst biopsy was performed with a noncontact 1.48 μ diode laser (OCTAX Microscience GmbH; Bruckberg, Germany) on day 5. Three to five trophectoderm (TE) cells were aspirated and loaded into a PCR tube with 2.5 μl 1× PBS as previously described [6,29,39]. At the same time, assisted hatching (AH) was performed for all embryos in the morphological assessment alone (Group B). Whole genomic amplification was performed with the SurePlex DNA amplification kit (BlueGnome, Cambridge, UK) and aCGH testing was performed using the 24sure protocol as described previously [6,39]. In brief, sample and control DNA (8 μl for each) were labeled with Cy3 and Cy5 fluorophores for 2–4 hours. The labeled DNA was re-suspended in dexsulphate hybridization buffer and then hybridized on the 24sure slides overnight. The hybridized slides were washed in 2× saline sodium citrate (SSC) plus 0.05% Tween-20 for 10 minutes and then in 1× SSC at room temperature for 10 minutes. The slides were washed in 0.1× SSC at 60°C for 5 minutes followed by the final wash in 0.1× SSC at room temperature for 1 minute. After centrifugation at 200 g for 3 minutes, the 24sure slides were scanned with a laser scanner at 10 μm (Agilent Technologies; Santa Clara, USA). BlueFuse Multi software (BlueGnome, Cambridge, UK) was used for analysis of microarray data on whole chromosomal gain and loss across all 24 chromosomes.

### Embryo selection for transfer and cryopreservation

In the morphology assessment plus aCGH screening group, only one euploid blastocyst with the best grade available was selected for fresh transfer to each patient and the remaining euploid blastocysts with good morphology (grade 3BB or above) were vitrified on day 6. In the morphology assessment alone group, a single blastocyst with the best grade available was selected for fresh transfer based on morphology assessment only and the remaining blastocysts with good morphology (grade 3BB or above) were vitrified using the same methods as Group A.

### Blastocyst vitrification and warming

Blastocysts from both Group A and Group B were vitrified using the Cryotip method as described elsewhere [41]. In brief, blastocysts were equilibrated in equilibration solution (ES) containing 7.5% DMSO, 7.5% ethyleneglycol, 20% synthetic serum substitute (SSS) or dextran serum supplement (DSS) for 9 to 12 minutes. They were then passed through four 20 μl drops of vitrification solution (VS) containing 15% DMSO, 15% ethyleneglycol, 0.5 M sucrose and 20% SSS or DSS. After washing in VS drops, individual blastocysts were loaded into the Cryotips within 90 seconds and plunged into liquid nitrogen immediately. For warming, the Cryotip was removed from liquid nitrogen and thawed in a 37°C water bath for about 3 seconds and the contents were released as a small drop. The Cryotip contents were then mixed with the thawing solution (TS) containing 1.0 M sucrose, 20% SSS or DSS in Medium-199 for 1 minute. The blastocysts were passed through two drops of dilution solution (DS) containing 0.5 M sucrose, 20% SSS or DSS. They were then passed through two drops of washing solution (WS) containing 20% SSS or DSS in Medium-199 before placing into blastocyst culture medium. After warming, one to two blastocysts were transferred to each patient depending on survival of the vitrified blastocysts in individual patients. No more than two blastocysts were transferred to each patient.

### Patient preparation for FET cycles

In both Group A and Group B, patients were treated using identical endometrial preparation protocols. In brief, norethindrone acetate and ethinyl estradiol (Loestrin®24Fe, Warner Chilcott) were used in combination with estradiol valerate (Delestrogen, JHP Pharmaceuticals). Preparation of the endometrium was initiated on day 4 of menstruation with Loestrin®24Fe (1 mg/day) until day 24 of the cycle. Then a total of six injections of 10 mg Delestrogen were administered 3 days after onset of menstruation, and the last injection of Delestrogen was given on the day of embryo transfer. Endometrial thickness was evaluated by ultrasound on days 9 to 12. Progesterone (50 mg/day) was given 3 days before embryo transfer. In cases of pregnancy, progesterone was administered for the first 9 weeks of pregnancy.

### Outcome measures and statistical analysis

The percentages of cryopreserved blastocysts and survival rates after warming were recorded and compared between Group A and Group B. Pregnancy outcomes and implantation rates per embryo transferred were also tabulated and compared between the two study groups. Differences between groups were assessed by Chi-square analysis or Fisher’s exact test. A difference of *p* <0.05 was considered statistically significant.

## Results

**Figure 1:**
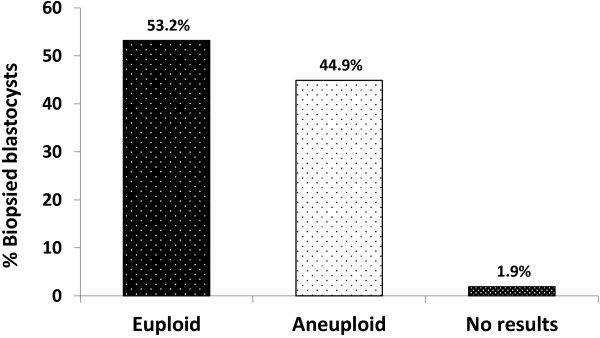
**A summary of aCGH results derived from biopsied blastocysts (n = 425) in the morphology assessment plus aCGH screening group.** No results = no results due to DNA amplification failure.

**Figure 2:**
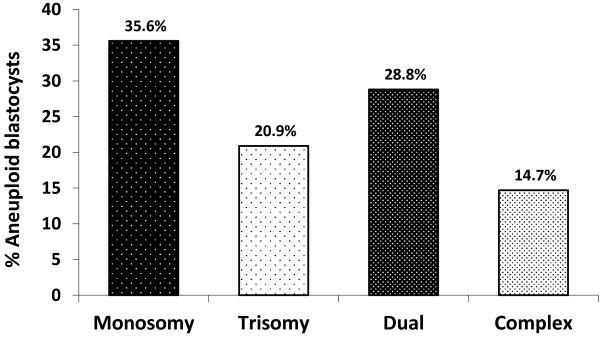
**Detail of aCGH results derived from aneuploid blastocysts (n = 191) in the morphology assessment plus aCGH screening group.** Monosomy = single chromosome loss; Trisomy = single chromosome gain; Dual = two chromosomal abnormality; Complex = three or more chromosomal abnormality.

First time IVF patients with good prognosis were randomized into two study groups: 55 patients in the morphology assessment plus aCGH screening (Group A) and 48 patients in the morphology assessment alone (Group B). The clinical and demographic features of the two study groups were similar. For patients in Group A, a total of 425 blastocysts were biopsied and analyzed with aCGH. The aCGH analysis revealed that 53.2% (226/425) of the blastocysts were euploid, 44.9% (191/425) were aneuploid and 1.9% (8/425) had no results due to DNA amplification failure (Figure 1). The percentages of each type of chromosomal abnormality detected in the aneuploid blastocysts were as follows: 35.6% (68/191) single chromosome loss (monosomy), 20.9% (40/191) single chromosome gain (trisomy), 28.8% (55/191) dual chromosomal abnormality and 14.7% (28/191) complex (three or more) chromosomal abnormality (Figure 2). Of the 191 aneuploid blastocysts, a total of 329 chromosome gains and losses involving all 24 chromosomes were detected by aCGH; 171 losses and 158 gains. While chromosomal abnormalities were detected in all chromosomes, disruptions involving chromosomes 15, 16, 21, 22 and X were observed most frequently. Abnormalities involving chromosomes 4, 5 and 6 were relatively uncommon.

Fresh single embryo transfer was performed on day 6 for all patients and the clinical outcome of the fresh transfer cycles was reported previously [6]. In summary, a single euploid blastocyst with the best morphology grade available was transferred to each patient in the morphology assessment plus aCGH screening (Group A). For patients in Group B (without aCGH screening) a single blastocyst with the best grade available was selected for fresh transfer based on morphology assessment alone (Table 1). The observed clinical pregnancy rate was significantly higher in Group A compared to Group B (70.9% vs. 45.8%, respectively; *p* = 0.017). A significant increase in ongoing pregnancy rates was also observed in Group A compared to Group B (69.1% vs. 41.7%, respectively; *p* = 0.009). There were no significant differences in miscarriage rates between Group A and Group B (2.6% vs. 9.1%, respectively; *p* >0.05). No twin pregnancies were identified in either group.

**Table 1:** Comparison of clinical outcomes of fresh SET between the morphology assessment plus aCGH screening (Group A) and the morphology assessment alone (Group B)

**Parameters**	**Group A**	**Group B**	***p *value**
Patients enrolled	55	48	
Patients with fresh SET	55	48	
Clinical pregnancy	39 (70.9%)	22 (45.8%)	0.017^a^
Ongoing pregnancy	38 (69.1%)	20 (41.7%)	0.009^a^
Miscarriage	1 (2.6%)	2 (9.1%)	0.597^b^

SET = single embryo transfer, ^a^by Chi-square analysis; ^b^by Fisher’s exact test.

As shown in Table 2, a total of 64 (28.3%) remaining euploid embryos with good morphology (grade 3 BB or above) were cryopreserved after fresh embryo transfer in Group A. For patients in Group B, 389 blastocysts were microscopically examined and a single blastocyst with the best grade available was selected for fresh transfer in each patient. A total of 157 (40.4%) blastocysts with good morphology (grade 3BB or above) were vitrified in this group. There was a significant difference in the percentage of cryopreserved blastocysts between Group A and Group B (28.3% vs. 40.4%, respectively; *p* = 0.003).

**Table 2:** A summary of IVF patients’ data for blastocyst cryopreservation following morphology assessment plus aCGH screening (Group A), or morphology assessment alone (Group B)

**Parameters**	**Group A**	**Group B**
Patients enrolled	55	48
Blastocysts evaluated by morphology	425	389
Blastocysts analyzed by aCGH	425	n/a
Euploid blastocysts identified by aCGH	226	n/a
% Cryopreserved blastocysts among non-transferred*	28.3%	40.4%

*Group A vs. Group B, *p* = 0.003 (by Chi-square analysis).

Data in Table 3 compare blastocyst survival rates after warming between the morphology assessment plus aCGH screening (Group A) and the morphology assessment alone (Group B). In Group A, 15 patients completed the FET cycles and a total of 22 blastocysts were thawed; 20 (90.9%) of the blastocysts survived after warming and were transferred (an average of 1.3 blastocysts per patient). In Group B, 23 patients completed the FET cycles and a total of 46 blastocysts were thawed. A total of 42 (91.3%) of the blastocysts survived after warming and were transferred (an average of 1.8 blastocysts per patients) in this group. There were no significant differences in blastocyst survival rates between the morphology assessment plus aCGH screening group and the morphology assessment alone group (90.9% vs. 91.3%, respectively; *p* >0.05).

**Table 3:** Comparison of blastocyst survival after vitrification and warming between the morphology assessment plus aCGH screening (Group A) and the morphology assessment alone (Group B)

**Parameters**	**Group A**	**Group B**	***p *value**
Patients completed FET cycles	15	23	
Thawed blastocysts	22	46	
Survived blastocysts	20 (90.9%)	42 (91.3%)	0.957*

*by Chi-square analysis; FET = frozen and thawed embryo transfer.

All FET patients were offered transfer of one or two blastocysts depending on survival of the vitrified blastocysts after warming. Data on frozen and thawed embryo transfer and pregnancy outcomes are summarized in Table 4. There was a significantly lower percentage of patients with double embryo transfer (DET) in the morphology assessment plus aCGH screening group compared to the morphology assessment alone group (33.3% vs. 82.6%, respectively; *p* = 0.006). There were no significant differences in clinical pregnancy rates between Group A and Group B (66.7% vs. 52.2%, respectively; *p* >0.05). The ongoing pregnancy rate in Group A was slightly higher than that of Group B (66.7% vs. 43.5%, respectively; *p* >0.05). However, the implantation rate per embryo transferred was significantly higher in the morphology assessment plus aCGH screening group when compared to the morphology assessment alone group (65.0% vs. 33.3%, respectively, *p* = 0.038). Moreover, there was no miscarriage observed in the morphology assessment plus aCGH screening group while a 16.7% miscarriage rate was recorded in the morphology assessment alone group (0% vs. 16.7%, respectively; *p* >0.05) although the difference was not statistically significant with the sample size. Additionally, there were no significant differences in twin pregnancy rates between Group A and Group B (30.0% vs. 16.7%, respectively; *p* >0.05).

**Table 4:** Comparison of clinical outcomes of the subsequent FET cycles between the morphology assessment plus aCGH screening (Group A) and the morphology assessment alone (Group B)

**Parameters**	**Group A**	**Group B**	***p *value**
**Patients completed FET cycles**	15	23	
**Patients with SET**	10 (66.7%)	4 (17.4%)	
**Patients with DET**	5 (33.3%)	19 (82.6%)	0.006^a^
**Clinical pregnancy**	10 (66.7%)	12 (52.2%)	0.583^a^
**Ongoing pregnancy (≥20 weeks GA)**	10 (66.7%)	10 (43.5%)	0.286^a^
**Implantation**	13 (65.0%)	14 (33.3%)	0.038^a^
**Twin pregnancy**	3 (30.0%)	2 (16.7%)	0.624^b^
**Miscarriage**	0	2 (16.7%)	0.481^b^

SET = single embryo transfer, DET = double embryo transer, GA = gestational age; ^a^by Chi-square analysis; ^b^by Fisher’s exact test.

## Discussion

The advance in embryo cryopreservation technology, particularly vitrification [41], has led to a substantial increase in cryopreservation of human embryos derived from IVF cycles [42-44] either for the purpose of reducing the incidence of ovarian hyper-stimulation syndrome (OHSS) or for future use in subsequent FET cycles. Since the first live birth was reported after a thawed embryo transfer with the traditional freezing protocols in 1984, frozen-thawed embryo transfer, particularly with vitrification and warming, has become an integral part of IVF treatment [45-50]. While embryo cryopreservation and subsequent FET continue to play an important role in assisted reproductive procedures with proven live birth [51-55], it still remains unknown exactly how new genome-wide molecular testing may affect the clinical outcome of this aspect of assisted reproductive treatment [39]. So far, clinical research has focused on application of such comprehensive chromosomal screening technology specifically for patients with a known translocation, repeated implantation failure or recurrent pregnancy loss [5,24-28,34], and more recently for some infertile patients [30,36] as well as first-time IVF patients with good prognosis [6]. Our previous reports have demonstrated that aCGH screening for first-time IVF patients with good prognosis significantly improves clinical and ongoing pregnancy rates in fresh embryo transfer cycles [6], while the number of embryos available for cryopreservation is sharply reduced in the same group of patients [39]. However, a prospective study on frozen and thawed embryo transfer as related to pregnancy and implantation outcomes following aCGH screening of embryos from IVF patients with such history (*i.e.*, first cycle IVF, age <35 and good prognosis) has not yet been conducted. While aCGH screening has been recently applied for selection of euploid embryo(s) for transfer in fresh and frozen embryo transfer cycles, this is the first prospective investigation into the impact of aCGH specifically on cryopreservation and implantation outcomes in subsequent FET cycles of first-time IVF patients with good prognosis.

Our study contributes new aCGH data on survival of frozen and thawed blastocysts and pregnancy outcomes in subsequent FET cycles of young, low-risk IVF patients. When embryo selection for cryopreservation was performed on the basis of morphology assessment plus aCGH screening, 90.9% of the cryopreserved blastocysts survived after warming. The survival rate of vitrified blastocysts in the morphology assessment plus aCGH screening group was similar to that of the morphology assessment alone group. Moreover, a significantly lower proportion of blastocysts was available for cryopreservation in Group A compared to Group B. However, a significantly higher implantation rate per embryo transfer was observed in the morphology assessment plus aCGH screening group compared to the morphological assessment alone group (65.0% vs. 33.3%, respectively, *p* = 0.038). Furthermore, there was no miscarriage observed in Group A while a 16.7% miscarriage rate was recorded in Group B (0% vs. 16.7%, respectively, *p* >0.05). Our data suggest that selection of euploid blastocysts for cryopreservation with aCGH screening for IVF patients may improve the efficiency of FET programs in several ways: 1) by selecting euploid blastocysts for cryopreservation to increase implantation rates per embryo transferred in subsequent FET cycles, 2) by eliminating aneuploidy blastocysts from the cryopreservation pool to decrease the risk of miscarriage following frozen and thawed embryo transfer and 3) by selective cryopreservation of euploid blastocysts to lower the overall cost associated with cryopreservation and storage, especially when such valuable blastocysts are vitrified and stored individually. A recent retrospective aCGH study comparing single thawed euploid embryo to routine age matched IVF patients undergoing blastocyst transfer has yielded similar pregnancy and implantation outcomes [30].

Our present investigation extends prior observations on the young and low-risk IVF population and finds conventional morphological criteria alone to be insufficiently accurate to select blastocysts for transfer and cryopreservation [6,39]. Because blastocysts in our control group (Group B) were selected by morphology assessment alone, they were cryopreserved without any aneuploidy screening, and therefore have an uncertain reproductive potential. The observed implantation rate per embryo transferred was significantly lower in the morphology assessment group without any aCGH testing when compared to the morphology assessment plus aCGH screening group. It has been well documented that transfer of aneuploid embryos in IVF patients results in implantation failure, miscarriage or birth of abnormal babies with serious medical problems [3,5,14]. The current study provides further evidence of clinical benefits for IVF patients with aCGH screening by eliminating the embryos with substantial chromosome abnormalities (including monosomy, trisomy, dual and complex aneuploidy) but with apparently “normal looking” morphology [3-6,39], which otherwise would have been destined for transfer and cryopreservation. Our data suggest that designating an embryo for cryopreservation without aCGH comprehensive chromosome screening may entail the preservation and storage of a reproductively incompetent embryo.

Several limitations of our investigation should be addressed. The frozen and thawed embryo transfer was carried out only in those patients who failed to conceive after fresh embryo transfer in both Group A and Group B since the patients who had become pregnant in the fresh transfer cycles were not yet ready for the FET cycles. Moreover, considering the negative pregnancy results in their fresh embryo transfer cycles, patients were offered to transfer one or two blastocysts depending on survival of the cryopreserved blastocysts after warming in individual patients. Some patients had only one blastocyst available for transfer while the other patients had more blastocysts available for transfer after warming. No more than two blastocysts were transferred to each patient. As a result, there were significantly more patients with double embryo transfer in the Group B than those in Group A (82.6% vs. 33.3%, respectively, *p* = 0.006). Additionally, we were not able to include a power analysis prior to this investigation because the actual incidence of embryo aneuploidy in first-time IVF patients with no risk factors is unknown, and the survival rates of embryos after vitrification and warming may vary from patient to patient.

## Conclusions

The present study demonstrates that aCGH screening of blastocysts prior to cryopreservation significantly increases implantation rates, and may reduce the risk of miscarriage in subsequent FET cycles. Our data suggest that designating an embryo for cryopreservation without aCGH comprehensive chromosome screening may entail the preservation and storage of a reproductively incompetent embryo. Further randomized clinical studies with a larger sample size are needed to validate these preliminary findings.

## Competing interest

The authors declare that they have no competing interests.

## Authors’ contributions

ZY and JL conceived the research, designed the study, and directed the aCGH analysis. ZY wrote the manuscript and organized the revisions. XL and KY were in charge of data mining and statistical analysis. SAS, JL, KY and RDS were the fertility specialists with oversight of the clinical program. All authors read and approved the final manuscript.
